# Outcomes of Gene Panel Testing for Sensorineural Hearing Loss in a Diverse Patient Cohort

**DOI:** 10.1001/jamanetworkopen.2022.33441

**Published:** 2022-09-27

**Authors:** Elizabeth N. Liao, Emily Taketa, Noura I. Mohamad, Dylan K. Chan

**Affiliations:** 1 Department of Otolaryngology–Head & Neck Surgery, University of California, San Francisco

## Abstract

**Importance:**

A genetic diagnosis can help elucidate the prognosis of hearing loss, thus significantly affecting management. Previous studies on diagnostic yield of hearing loss genetic tests have been based on largely homogenous study populations.

**Objectives:**

To examine the diagnostic yield of genetic testing in a diverse population of children, accounting for sociodemographic and patient characteristics, and assess whether these diagnoses are associated with subsequent changes in clinical management.

**Design, Setting, and Participants:**

This retrospective cohort study included 2075 patients seen at the Children’s Communications Clinic, of whom 517 completed hearing loss gene panel testing between January 1, 2015, and November 1, 2021, at the University of California, San Francisco Benioff Children’s Hospital system. From those 517 patients, 426 children with at least 2 audiograms were identified and analyzed. Data were gathered from November 2021 to January 2022 and analyzed from January to February 2022.

**Main Outcomes and Measures:**

The measures of interest were sociodemographic characteristics (age at testing, gender, race and ethnicity, primary language, and insurance type), hearing loss characteristics, and medical variables. The outcome was genetic testing results. Variables were compared with univariate and multivariable logistic regression.

**Results:**

Of the 2075 patients seen at the Children’s Communications Clinic, 517 (median [range] age, 8 [0-31] years; 264 [51.1%] male; 351 [67.9%] from an underrepresented minority [URM] group) underwent a hearing loss panel genetic test between January 1, 2015, and November 1, 2021. Among those 517 patients, 426 children (median [range] age, 8 [0-18] years; 221 [51.9%] male; 304 [71.4%] from an URM group) with 2 or more audiograms were included in a subsequent analysis. On multivariable logistic regression, age at testing (odds ratio [OR], 0.87; 95% CI, 0.78-0.97), URM group status (OR, 0.29; 95% CI, 0.13-0.66), comorbidities (OR, 0.27; 95% CI, 0.14-0.53), late-identified hearing loss (passed newborn hearing screen; OR, 0.27; 95% CI, 0.08-0.86), and unilateral hearing loss (OR, 0.04; 95% CI, 0.005-0.33) were the only factors associated with genetic diagnosis. No association was found between genetic diagnosis yield and other sociodemographic variables or hearing loss characteristics. Patients in URM and non-URM groups had statistically similar clinical features. A total of 32 of 109 children (29.4%) who received a genetic diagnosis received diagnoses that significantly affected prognosis because of identification of syndromic or progressive sensorineural hearing loss or auditory neuropathy spectrum disorder relating to otoferlin.

**Conclusions and Relevance:**

This cohort study’s findings suggest that genetic testing may be broadly useful in improving clinical management of children with hearing loss. More research is warranted to discover and characterize diagnostic genes for those who have been historically underrepresented in research and medicine.

## Key Points

**Question:**

What is the association between rates of genetic diagnosis and sociodemographic and clinical characteristics in children with sensorineural hearing loss, and how often are these diagnoses associated with changes in clinical management?

**Findings:**

In this cohort study of 426 children, those who were older, had more medical comorbidities, identified as being from an underrepresented minority group, had late-identified hearing loss (passed newborn hearing screen), and/or had unilateral hearing loss were less likely to receive a genetic diagnosis; all other sociodemographic and audiologic factors were not significantly associated with genetic diagnosis rates. Thirty-two of 109 children (29.4%) received genetic diagnoses that was associated with subsequent changes in clinical management.

**Meaning:**

These findings may help improve patient counseling and shared decision-making for patients and families who are deciding whether to obtain genetic testing.

## Teaser

This retrospective study of children with sensorineural hearing loss examines whether genetic testing diagnoses are associated with sociodemographic characteristics and clinical management.

## Introduction

Hearing loss (HL) is the most common congenital sensory disorder in children, with an estimated incidence of neonatal HL of approximately 0.1%,^1^ which increases to 1 in 300 children by 9 years of age. Hearing loss can be attributable to a variety of factors, including genetic causes, trauma, congenital infections, drug toxic effects, and syndromic HL.^2,3,4,5^ Determining the cause of HL helps clinicians and patients and their families decide the best medical and audiologic intervention for the child’s HL. For instance, HL attributable to congenital cytomegalovirus infection has a high risk of progression, so clinicians and families may decide to proceed with early cochlear implantation to optimize speech and language outcomes,^5^ whereas patients with Usher syndrome have both progressive vision and HL and are closely followed by ophthalmologists and otolaryngologists.^2^ A genetic diagnosis can similarly help elucidate the type and progression of HL, thus significantly affecting management.^6^

During the past decade, genetic testing has become a cornerstone of diagnostic workup for children with HL. Causative variants in more than 150 genes have been identified so far, including *GJB2* (OMIM 220290), *SLC26A4* (OMIM 605646), *OTOF* (OMIM 603681), *CDH23* (OMIM 601067), and *STRC* (OMIM 606440).^6^ Understanding the causative genes for a child’s HL can provide important prognostic information; many genes and variants have characteristic clinical phenotypes and can be broadly categorized into those principally associated with nonsyndromic stable sensorineural HL (SNHL), nonsyndromic progressive SNHL, and syndromic SNHL. Nonsyndromic stable SNHL genes include *GJB2, STRC,* and *OTOG*^6,7,8,9,10^; nonsyndromic progressive SNHL genes include *MIR96* (OMIM 611606), *TECTA* (OMIM 602574)*,* and *SLC26A4*^10,11,12,13,14,15,16,17,18,19^; syndromic genes include *ALMS* (OMIM 203800) for Alstrom syndrome, *EYA1* (OMIM 601653) for branchiootorenal syndrome, *WFS1* (OMIM 606201) and *WFS2* (OMIM 604928) for Wolfram syndrome, and *USH2A* (OMIM 608400) and *MYO7A* (OMIM 276903) for Usher syndrome^20,21,22,23,24,25^; other genes provide other specific clinical prognostic information, such as *OTOF*, which causes auditory neuropathy spectrum disorder with excellent prognosis with cochlear implantation.^26^

The diagnostic yield for HL gene panel testing has been reported to range from 12.7% to 64.3%.^27,28,29,30,31,32,33,34,35,36,37,38,39,40^ These rates have been based on research studies with largely homogenous study populations. Most had participants of European or East Asian origin; only 3 studies^28,39,41^ included people of African origin. In addition, only 3 studies^37,39,41^ broke down diagnostic rates by HL characteristics. Sloan-Heggen et al^41^ described HL based on laterality, severity, and age at onset; Florentine et al^39^ described age at onset, progression, laterality, and severity; and Yuan et al^37^ described age at onset, severity, and laterality. However, conclusions on clinical factors associated with genetic diagnostic rate have been severely limited, because these studies did not adjust for demographic factors that are significantly associated with diagnostic rate, especially race and ethnicity. Genetic testing in a more diverse population is critical for equitable management of children with HL.

We sought to address this gap by describing the diagnostic yield of genetic testing in a diverse population of children, critically accounting for both sociodemographic and HL characteristics. Understanding the true association of demographic and clinical factors with genetic diagnosis rate will facilitate the clinical decision-making process for all children with SNHL. We also sought to understand how often a genetic diagnosis is associated with clinical management in our cohort.

## Methods

### Study Design and Population

We performed a retrospective cohort study patients aged 0 to 18 years with SNHL with at least 2 audiograms and who completed HL gene panel testing (GeneDX)^42^ between January 1, 2015, and November 1, 2021. Patients were included if they were ever seen at the Children’s Communications Clinic of University of California, San Francisco (UCSF)-Benioff Children’s Hospital and a HL gene panel test had resulted by November 1, 2021. Patients were excluded if they had no or only 1 audiogram in their electronic medical record or if gene panel testing was done at or after 18 years of age. Data were gathered from November 2021 to January 2022 and analyzed from January 2022 to February 2022. All families provided written informed consent for testing. This study was approved by the University of California, San Francisco and followed the Strengthening the Reporting of Observational Studies in Epidemiology (STROBE) reporting guideline.

### Sociodemographic Variables

Patient gender, ethnicity, race, primary home language, and insurance type were based on parent self-report on clinic registration, coded into the electronic medical record, and manually extracted from these coded fields by a researcher (E.N.L.) who was blinded to genetic diagnosis status. Patients were classified as being from an underrepresented minority (URM) group based on the National Institutes of Health’s working definition of underrepresented minority groups^42^ (ie, those who self-report as Black, Hispanic, Native American or Alaska Native, Native Hawaiian or other Pacific Islander, or ≥2 races when ≥1 are from the forementioned categories were defined as being from a URM group). Patients were categorized as other race or ethnicity if they did not identify as Asian, Black, Hispanic, or White; patients could otherwise identify as more than 1 race or ethnicity.

### Audiologic and Medical Variables

Audiologic and medical data were manually collected from otolaryngology, audiology, and anesthesiology notes by a researcher (E.N.L.) who was blinded to genetic diagnosis status. For audiograms, we collected the pure tone averages of the first-ever diagnostic hearing test, which was either an audiogram, auditory brainstem response, or auditory steady-state response. We also collected the pure tone averages of the latest hearing test, which was the latest audiogram, auditory brainstem response, or auditory steady-state response. If the first-ever diagnostic and/or latest hearing test results were not available, then we used the next available hearing test. Pure tone averages were calculated based on hearing thresholds for pure tone frequencies of 500, 1000, 2000, and 4000 Hz; speech detection or reception thresholds were recorded if these data were not available; and sound fields were used only if neither pure tone averages nor speech detection or reception thresholds were available.

Patients were categorized into early identified, late identified, and late discovered based on how they were identified with HL: early identified indicates that the patient failed the newborn hearing screen in 1 or both ears, late identified indicates the patient passed the newborn hearing screen in both ears and was found to have HL later in life, and late discovered indicates that the newborn hearing screen was not performed or unknown and the child was found to have HL later in life. Progression of HL was defined as a greater than 15 dB increase in pure tone average, speech detection or reception thresholds, or sound field between the 2 hearing tests.

The pediatric-specific American Society of Anesthesiologists (ASA) physical classification system^43^ and *International Statistical Classification of Diseases and Related Health Problems, Tenth Revision* (*ICD-10*) codes were used as a proxy for the presence and severity of medical comorbidities. The ASA scores were manually extracted from anesthesia notes (E.N.L.). The *ICD-10* codes were extracted from the patient’s problem list in the electronic medical record via structured data query of the electronic medical record.

### Genetic Testing Variables

California state public insurance initiated coverage of HL gene panel testing in 2017. We initiated point-of-care sample collection in the otolaryngology and audiology clinics, enabling efficient and equitable access to testing across our entire patient population. All families received pretest and posttest genetic counseling.

Hearing loss gene panel testing uses targeted gene capture and massive parallel sequencing to sequence nuclear and mitochondrial genes that are causative for SNHL. In 2015, a total of 130 genes were tested in this panel; in 2021, a total of 146 nuclear genes and 6 variants in 4 mitochondrial genes were tested. Each variant is classified as benign, likely benign, variant of uncertain significance, likely pathogenic, or pathogenic based on the American College of Medical Genetics and Genomics 2015 Guidelines.^44^ Each variant was further described by mode of inheritance (autosomal recessive, autosomal dominant, both, other) and zygosity (heterozygous or homozygous).

Patients received a positive genetic diagnosis based on the variant designation, mode of inheritance, and zygosity.^45^ These data were manually collected directly from the genetic testing reports accessed through the genetic testing portal, independent of the electronic medical record, by researchers (E.T. and N.I.) who were blinded to the study hypothesis and all other study data. If a patient’s genetic test results were insufficient for diagnosis, gene panel test results of the patient’s parents were manually reviewed to determine whether variants were inherited in from 1 or both parents. For instance, a variant that is pathogenic, autosomal recessive, and simple heterozygous was not diagnostic; a variant that is pathogenic, autosomal dominant, and simple heterozygous was diagnostic; and 2 variants of the same gene that were pathogenic, autosomal recessive, and compound heterozygous were diagnostic if 1 variant was inherited from one parent and the other variant was inherited from the other parent (trans-configuration) and nondiagnostic if both variants were inherited from a single parent (cis-configuration).

### Statistical Analysis

A total of 230 children (54.0%) did not have ASA scores because they did not have a procedure that necessitated evaluation by an anesthesiologist; 98 children (23.0%) did not have any *ICD-10* codes in their electronic medical record. Two children (0.4%) had no insurance listed; there were no missing data for the other variables. Descriptive statistics were used to describe the cohort: numbers (percentages) were used for categorical variables, and medians (ranges) were used for continuous variables because the variables did not follow a normal distribution. Variables were compared with logistic regression. Adjusted odds ratios (ORs) and 95% CIs were calculated using multiple logistic regression. All analysis was completed with Stata software, version 17.0 (SAS Institute Inc).

## Results

### Study Population

Of the 2075 patients seen at the Children’s Communications Clinic, 517 (median [range] age, 8 [0-31] years; 264 [51.1%] male; 351 [67.9%] from a URM group) underwent a HL panel genetic test from January 1, 2015, to November 1, 2021, of whom 426 were children with 2 or more audiograms (median [range] age, 8 [0-18] years; 221 [51.9%] male; 304 [71.4%] from a URM group). The demographic data of the children (including race and ethnicity as well as primary language), comorbidities, and HL characteristics, compared with the general population of children with SNHL seen at the clinic, are given in Table 1. This population of tested children was considerably diverse: 351 (67.9%) self-identified as being from a URM group, 365 (70.6%) had public insurance, and 202 (39.1%) did not speak English as their primary language. The demographic and clinical profile of the tested cohort was also consistent during the last 3 years of the study, when most patients were tested (eTable 1 in the Supplement). After excluding children with fewer than 2 audiograms and those who were 18 years or older at testing, 426 children were included in the subsequent analysis. This subpopulation was representative of the larger cohort of all tested children in terms of demographic data, comorbidities, and HL characteristics (Table 1). The diagnostic yield in this cohort was 25.6% (n = 109).

**Table 1.** Description of the Study Cohort^a^

Characteristic	All patients seen at the Children’s Communications Clinic (N = 2075)	All patients who received a genetic test (n = 517)	Study cohort (n = 426)
Age, median (range), y	12 (0 to 43)	8 (0 to 31)	8 (0 to 18)
Sex			
Male	990 (47.7)	264 (51.1)	221 (51.9)
Female	937 (45.2)	252 (48.7)	204 (47.9)
Transgender	2 (0.0)	1 (0.2)	1 (0.2)
Unknown	146 (7.0)	0	0
URM status			
URM	1254 (60.4)	351 (67.9)	304 (71.4)
Non-URM	602 (29.0)	158 (30.6)	116 (27.2)
Unknown or declined to state	219 (10.6)	8 (1.6)	6 (1.4)
Race and ethnicity^b^			
Asian	328 (13.8)	96 (18.6)	73 (17.1)
Black	108 (5.2)	37 (7.2)	34 (8.0)
Hispanic	740 (35.7)	226 (43.7)	195 (45.8)
White	543 (26.2)	134 (25.9)	106 (24.9)
Other^c^	438 (21.1)	91 (17.6)	76 (17.8)
Unknown	219 (10.6)	0	0
Primary language			
English	1278 (61.6)	315 (60.9)	256 (60.1)
Spanish	432 (20.8)	128 (24.8)	113 (26.5)
Mandarin, Cantonese	76 (3.7)	22 (4.3)	14 (3.3)
American Sign Language	56 (2.7)	14 (2.7)	9 (2.1)
Other language	71 (3.4)	38 (7.4)	34 (8.0)
Unknown	162 (7.8)	0	0
Insurance			
Private	728 (35.1)	147 (28.4)	113 (26.5)
Public	969 (46.7)	365 (70.6)	311 (73.0)
None	0	5 (1.0)	2 (0.5)
Unknown	378 (18.2)	0	0
Comorbidities			
No. of *ICD-10* codes, median (range)	3 (0 to 72)	2 (0 to 40)	2 (0 to 40)
No. of *ICD-10* organ systems involved, median (range)	2 (0 to 17)	1 (0 to 10)	1 (0 to 10)
ASA score, mode (range)	2 (1 to 5)	2 (1 to 4)	2 (1 to 4)
Audiologic data			
Time of identification or discovery			
Early identification (failed NHS)	723 (34.8)	194 (37.5)	176 (41.3)
Late identification (passed NHS)	418 (20.1)	149 (28.8)	130 (30.5)
Late discovery (unknown NHS)	209 (10.1)	152 (29.4)	120 (28.2)
Unknown	725 (34.9)	22 (4.3)	0
Characterization of HL			
Progressive	502 (24.2)	101 (19.5)	99 (23.2)
Stable	143 (6.9)	339 (65.6)	327 (76.7)
Unknown	1430 (69.0)	77 (14.9)	0
Laterality of HL			
Unilateral	348 (16.8)	103 (19.9)	90 (21.1)
Bilateral	1021 (49.2)	385 (74.4)	336 (78.9)
Unknown	705 (34.0)	29 (5.6)	0
Type of HL^b^			
Sensorineural	1016 (48.96)	421 (81.4)	340 (79.8)
Conductive	81 (3.9)	17 (3.3)	16 (3.8)
Mixed	94 (4.5)	37 (7.2)	32 (7.5)
Neural	54 (2.6)	19 (3.7)	18 (4.2)
Unspecified	35 (1.7)	38 (7.4)	36 (8.5)
Unknown	838 (40.4)	0	0
Severity of HL			
PTA better ear, median (range), dB	31 (−1 to 121)	33 (0 to 120)	33 (0 to 120)
PTA worse ear, median (range), dB	53 (1 to 125)	45 (5 to 120)	45 (5 to 120)
Genetic diagnosis			
Received a genetic diagnosis	NA	136 (26.3)	109 (25.6)
Did not receive a genetic diagnosis	NA	381 (73.7)	317 (74.4)

Abbreviations: ASA, American Society of Anesthesiologists; HL, hearing loss; *ICD-10, International Classification of Diseases, 10th Revision*; NA, not applicable; NHS, newborn hearing screen; PTA, pure tone average; URM, underrepresented minority.

^a^ Data are presented as number (percentage) of patients unless otherwise indicated.

^b^ Does not total 100% because patients can identify as more than 1 category.

^c^ Patients were categorized as other race or ethnicity if they did not identify as Asian, Black, Hispanic, or White; patients could otherwise identify as more than 1 race or ethnicity.

### Diagnostic Yield by Audiologic Characteristics

On univariable analysis, those who received a genetic diagnosis were younger than those who did not receive a genetic diagnosis (OR, 0.92; 95% CI, 0.88-0.96) and had a lower ASA score (ie, were healthier) (OR, 0.36; 95% CI, 0.20-0.62). No statistical association was found with receiving a genetic diagnosis and number of *ICD-10* codes. Genetic diagnosis was less likely among children who identified as being from a URM group (OR, 0.38; 95% CI, 0.24-0.60) or Hispanic (OR, 0.46; 95% CI, 0.29-0.73), had late-identified HL (OR, 0.60; 95% CI, 0.36-0.99), or had unilateral HL (OR, 0.07; 95% CI, 0.02-0.24). Those who identified as Asian (OR, 3.46; 95% CI, 2.00-5.96) or had early identified HL (OR, 2.02; 95% CI, 1.29-3.13) were significantly more likely to receive a genetic diagnosis. Gender, other individual racial or ethnic categories, primary language, insurance type, late-discovery HL, type of HL, progression, severity of HL in the worse hearing ear were not statistically associated with diagnostic yield (Table 2).

**Table 2.** Diagnostic Yield of Genetic Testing for Hearing Loss^a^

Characteristic	Genetic diagnosis (n = 109)	No genetic diagnosis (n = 317)	Pairwise OR (95% CI)	Adjusted OR (95% CI)^b^
Age, median (range), y	6 (0-17)	9 (0-18)	0.92 (0.88-0.96)^c^	0.87 (0.78-0.97)^c^
Male sex	54 (49.5)	167 (52.7)	0.86 (0.56-1.33)	NA
Underrepresented minority group	61 (55.9)	243 (76.7)	0.38 (0.24-0.60)^c^	0.29 (0.13-0.66)^c^
Asian	32 (29.4)	34 (10.7)	3.46 (2.00-5.96)	NA
Black	6 (5.5)	28 (8.8)	0.60 (0.24-1.49)	NA
Hispanic	35 (32.1)	160 (50.4)	0.46 (0.29-0.73)	NA
White	23 (21.1)	83 (26.2)	0.75 (0.45-1.27)	NA
Other^d^	21 (19.3)	55 (17.3)	1.14 (0.65-1.99)	NA
English as primary language	67 (61.5)	189 (59.6)	0.92 (0.59-1.45)	NA
Public insurance	75 (68.8)	236 (74.4)	0.84 (0.53-1.33)	NA
Comorbidities				
*ICD-10* codes, median (range)	2 (0-21)	2 (0-40)	0.95 (0.9-1.01)	NA
ASA score, median (range)	1.5 (1-3)	2 (1-4)	0.36 (0.20-0.62)^c^	0.27 (0.14-0.53)^c^
Audiologic data				
Early identification (failed NHS)	59 (54.1)	117 (36.9)	2.02 (1.29-3.13)^c^	0.37 (0.12-1.17)
Late identification (passed NHS)	25 (22.9)	105 (33.1)	0.60 (0.36-0.99)^c^	0.27 (0.08-0.86)^c^
Late discovery (unknown NHS)	25 (22.9)	95 (30.0)	0.69 (0.42-1.15)	NA
Characterization of HL				NA
Unilateral HL	3 (3)	87 (27)	0.07 (0.02-0.24)^c^	0.04 (0.005-0.33)^c^
Sensorineural HL	90 (82.6)	250 (78.9)	1.27 (0.72-2.23)	NA
Neural HL	3 (2.7)	15 (4.7)	0.57 (0.16-2.01)	NA
Severity of HL				
PTA of better hearing ear, median (range), dB	41 (8-115)	30 (0-120)	1.01 (1.00-1.02)	NA
PTA of worse hearing ear, median (range), dB	45 (10-120)	45 (5-120)	1.00 (0.99-1.01)	NA

Abbreviations: ASA, American Society of Anesthesiologists; HL, hearing loss; *ICD-10, International Classification of Diseases, 10th Revision*; NA, not applicable; NHS, newborn hearing screen; OR, odds ratio; PTA, pure tone average; URM, underrepresented minority.

^a^ Data are presented as number (percentage) of patients unless otherwise indicated.

^b^ Adjusted ORs (95% CIs) were calculated using multiple logistic regression using the variables indicated (age at testing, URM status, median ASA score, late identification, and laterality).

^c^ Statistically significant (*P* < .05).

^d^ Patients were categorized as other race or ethnicity if they did not identify as Asian, Black, Hispanic, or White; patients could otherwise identify as more than 1 race or ethnicity.

In multivariable analysis (inclusive of the variables that were significant in univariate analysis: age, URM status, ASA score, early identification, late identification, and unilateral HL [identifying as Hispanic or Asian was thought to be adequately included under the URM variable]), genetic diagnosis was less likely in those who identified as being in a URM group (aOR, 0.29; 95% CI, 0.13-0.66), in those with more comorbidities (aOR, 0.27; 95% CI, 0.14-0.53), for those with unilateral HL (aOR, 0.04; 95% CI, 0.005-0.33), for older children (aOR, 0.87; 95% CI, 0.78-0.97), and for late-identified HL (aOR, 0.27; 95% CI, 0.08-0.86). Early identification was not found to be significant in multivariable analysis (Table 2).

Patients who identified as URM and non-URM had statistically identical clinical features; there was no statistically significant difference in age at testing, ASA score, type of identification of HL, or type or laterality of HL (Table 3). However, those who identified as URM were more likely to have public insurance (OR, 2.90; 95% CI, 1.84-4.57). Insurance status in turn was not statistically associated with number of *ICD-10* codes (OR, 1.02; 95% CI, 0.97-1.06) or ASA score (OR, 1.31; 95% CI, 0.80-2.16).

**Table 3.** Clinical Characteristics of Patients Identifying and Not Identifying as Being in a URM Group^a^

Characteristic	URM group (n = 304)	Non-URM group (n = 116)	Pairwise OR (95% CI)
Age, median (range), y	8 (0-18)	7 (0-18)	1.01 (0.97-1.06)
Male sex	154 (50.6)	64 (55.2)	0.86 (0.56-1.31)
English as primary language	175 (57.6)	77 (66.4)	1.46 (0.93-2.28)
Public insurance	242 (79.6)	63 (54.3)	2.90 (1.84-4.57)^b^
Comorbidities, median (range)			
*ICD-10* codes	2 (0-40)	2 (0-24)	0.99 (0.96-1.04)
ASA score	2 (1-4)	2 (1-3)	1.31 (0.82-2.09)
Audiologic data			
Early identification (failed NHS)	128 (42.1)	47 (40.5)	1.04 (0.67-1.62)
Late identification (passed NHS)	89 (29.3)	39 (33.6)	0.82 (0.52-1.29)
Late discovery (unknown NHS)	87 (28.6)	30 (25.9)	1.17 (0.72-1.90)
Characterization of HL			
Unilateral HL	66 (21.7)	21 (18.1)	1.25 (0.73-2.16)
Sensorineural HL	248 (81.6)	88 (75.9)	1.41 (0.84-2.36)
Neural HL	14 (4.6)	4 (3.4)	1.35 (0.44-4.19)
Severity of HL			
PTA of better hearing ear, median (range), dB	34 (0-120)	28 (0-116)	1.01 (1.00-1.01)^b^
PTA of worse hearing ear, mean (SD) dB	46 (5-120)	44 (5-120)	1.01 (1.00-1.01)^b^

Abbreviations: ASA, American Society of Anesthesiologists; HL, hearing loss; *ICD-10, International Classification of Diseases, 10th Revision*; NHS, newborn hearing screen; OR, odds ratio; PTA, pure tone average; URM, underrepresented minority.

^a^ Data are presented as number (percentage) of patients unless otherwise indicated.

^b^ Statistically significant (*P* < .05).

### Diagnostic Genes by Race and Ethnicity and Type of HL

We categorized the 109 patients who received a genetic diagnosis by race and ethnicity and their diagnostic genes by associated phenotype (Table 4). Overall, 77 children (70.6%) who received genetic diagnoses had causative genes or variants identified that were associated with nonsyndromic, mostly stable SNHL, primarily *GJB2*. A total of 32 of 109 children (29.4%) received genetic diagnoses that significantly affected clinical management (7.5% of the entire study cohort). These genes revealed novel prognostic information on progression, syndromic association, or cochlear implant expectations: 9 had variants associated with nonsyndromic progressive SNHL, 23 had syndromic HL variants, and 2 had variants in otoferlin (which is associated with auditory neuropathy spectrum disorder^26^). Both URM and non-URM children had similar likelihoods of receiving 1 of these significant genetic diagnoses.

**Table 4.** Number of Individuals With Diagnostic Genes and Variants, Categorized by Race, Ethnicity, and Type of Hearing Loss^a^

Diagnostic gene	Hispanic	Asian	Black	White	Other
Nonsyndromic, mostly stable SNHL					
*GJB2*^7^					
c.35del	9	1	1	11	6
c.109 G>A	2	24	0	1	5
Other	8	4	0	4	1
*GJB2, GJB6*^7^	2	0	0	0	0
*OTOG*^8^	1	0	0	0	1
*OTOGL*^9^	2	0	0	0	1
*STRC*^10^	0	0	0	1	0
*STRC, CATSPER2*^10^	3	0	1	2	0
Nonsyndromic, mostly progressive SNHL					
*KCNQ4*^15^	0	0	1	0	1
*MIR96*^11^	0	0	0	0	1
*MYO15A*^16^	1	0	0	0	0
*POU3F4*^17^	1	0	1	0	0
*PTPRQ*^18^	0	0	0	0	0
*SLC26A4*^13^^a^	0	1	1	1	0
*TMPRSS3*^19^	0	0	0	1	0
Syndromic SNHL					
*ADGRV1*^22^ (Usher syndrome)	0	0	0	0	1
*ALMS1*^21^ (Alstrom syndrome)	1	0	0	0	0
*CDH23*^14,25^ (Usher syndrome)^b^	0	0	1	1	1
*EYA1*^23^ (branchiootorenal)	1	0	0	0	0
*MITF*^24^ (Waardenburg syndrome)	0	0	0	0	1
*MYO7A*^22^ (Usher syndrome)	0	1	0	1	0
*PDZD7*^22^ (Usher syndrome)	1	0	0	0	0
*SLC26A4*^13^^c^	0	1	1	1	0
*SOX10*^24^ (Waardenburg syndrome)	0	0	0	1	1
*STRC, CATSPER2* (deafness-infertility syndrome)^d^	2	0	0	2	0
*USH1C*^22^ (Usher syndrome)	0	0	0	0	2
*USH2A*^22^ (Usher syndrome)	2	1	0	2	1
Other					
*OTOF*^26^	1	1	0	0	0

Abbreviation: SNHL, sensorineural hearing loss.

^a^ Only children in the study cohort (n = 426) were included. Some of the genes listed as stable SNHL have very slowly progressive hearing loss. The genes we categorized as progressive were the ones that are more rapidly progressive and thus would be clinically relevant.

^b^ *CDH23* variants are associated with either nonsyndromic hearing loss or Usher syndrome. Our patients had the variants associated with Usher syndrome.

^c^ *SLC26A4* variants are associated with both syndromic and nonsyndromic progressive SNHL.

^d^ Males with a homozygous multigene deletion involving *STRC* and *CATSPER* have increased risk of deafness-infertility syndrome.^10^

Twenty-four diagnostic genes were found in our study cohort. The most common diagnostic gene by far was *GJB2*, a gene that encodes for connexin 26, a major protein found in gap junction protein in the cochlea. The most prevalent diagnostic variant was different across races and ethnicities: Asian patients were more likely to have the c.109 G>A variant, White patients were more likely to have the c.35del, and Hispanic patients were almost equally likely to have the c.35del variant as any variant aside from c.109 G>A. The second most common cause was a multigene deletion that involved *STRC* and *CATSPER2* (OMIM 607249); in males, this is associated with deafness-infertility syndrome.^10^ The next most common variants were in *USH2A* and *STRC*. More detailed categorization by diagnostic genes and variants of all 517 children can be found in eTable 2 in the Supplement.

## Discussion

In this study, we demonstrated that the overall diagnostic yield in this diverse population was 25.6%. Variables associated with decreased diagnostic yield were age at testing, unilateral HL, identification as being from a URM group, late-identified HL, and medical comorbidities. We also found that 29.4% of children who received a genetic diagnosis (7.5% of the entire study cohort) received diagnoses that significantly affected prognosis because of identification of syndromic or progressive SNHL or auditory neuropathy spectrum disorder relating to otoferlin. For instance, 4 male children had homozygous multigene deletion that involved *STRC* and *CATSPER2,* placing them at increased risk of deafness-infertility syndrome, which causes HL and infertility. Some children were also diagnosed with Usher syndrome, which is associated with HL and vision loss.

The cohort studied here is unique in its diversity and completeness of clinical data, with 71.4% identifying as belonging to a URM group. This percentage aligns with the overall patient population within our clinic and contrasts prior studies^27,28,29,30,31,32,33,34,35,36,37,38,39,40^of HL genetics, which have largely excluded these populations. The preponderance of individuals from URM groups likely contributes to our low diagnostic yield, because Hispanic and Black children are significantly less likely to receive a genetic diagnosis.^39^ Children from URM groups undergo genetic testing at significantly lower rates; clinicians may have different clinical indexes of suspicion for genetic causes of HL for URM vs non-URM children that drove their thresholds to obtain genetic testing. This bias would manifest as differences in clinical characteristics between URM and non-URM children who undergo genetic testing and may also have previously artificially overestimated the diagnostic rate in URM children and underestimated the disparity between diagnostic rate between URM and non-URM children. In our cohort, however, the clinical features of URM vs non-URM children were statistically identical, suggesting that there was minimal bias in how clinicians decided who underwent genetic testing. Thus, this finding suggests that we are accurately assessing the diagnostic yield in URM vs non-URM children.

Our audiologic conclusions agree with the existing literature. Although Sloan-Heggen et al,^41^ Florentine et al,^39^ and Yuan et al^37^ described HL based on different characteristics, only Sloan-Heggen et al^41^ tested these characteristics against diagnostic yield rates, showing that patients with unilateral HL and late-onset HL were significantly less likely to receive a diagnosis. This previous study,^41^ however, did not adjust for race or ethnicity in a multivariable model. Our study found that URM patients had similar presentations for HL, whether it be early identified, late identified, or late discovered. Children with late-identified and late-discovered HL present to the clinician similarly after birth, but our findings suggest that there may be different causes for late-identified vs late-discovered HL, because late-identified HL was statistically associated with decreased diagnostic yield, but late-discovered HL was not statistically associated. More work is warranted to screen children for HL if they did not receive a newborn hearing result and/or to check up on children with failed newborn hearing screen results.

### Limitations

This study has limitations. Because this is a retrospective study, we were unable to determine causality, only associations. Our audiologic data were heterogeneous; because of the range of ages included, we used a variety of audiologic data to estimate levels and progression, including estimated thresholds from auditory brainstem response recordings, speech reception and detection thresholds, or sound field thresholds, as well as ear-specific pure-tone thresholds. In addition, there is currently no consensus definition for progression, with definitions ranging from a 20-dB decrease in pure tone average thresholds to a 10-dB difference in 1 frequency.^46,47^ Updates in the genes tested in the gene panel may have affected the overall diagnostic yield; however, the anticipated effect is small, because these new genes are very rare and only 3 children received a diagnosis from this subset of genes.

Finally, ASA and *ICD-10* codes were the best available codable factors for medical comorbidities in our retrospective study; children with ASA scores and higher numbers of *ICD-10* codes were more likely to be medically complex. Generalizability may be limited because many children did not have an ASA score and/or *ICD-10* codes, and *ICD-10* codes are dependent on clinician entry into the electronic medical record. This limitation may explain why we found statistical associations between receiving a genetic diagnosis and ASA scores but not with number of *ICD-10* codes.

Children with late-onset HL (HL that began after the newborn period) can be difficult to distinguish clinically from children with late-identified HL, who comprise a complex category of children with congenital HL that should have been detected at birth but was not (either because of the absence of or loss to follow-up after newborn hearing screening) or having a level of hearing loss at birth that was not detected by newborn hearing screening. We chose to categorize our children into early identified vs late identified because this is how children present to clinicians based on their knowledge of newborn hearing screening results and age at identification of HL. In this way, we aim to make our results relevant from the perspective of clinicians seeking to counsel families on the likelihood of diagnostic results from genetic testing.

In this study, we used race and ethnicity as a proxy for genetic ancestry and admixture mapping. We recognize that this may have limited our results, given that race and ethnicities are social constructs, have been inconsistently applied to describe populations, and are not the sole contributors to phenotypic diversity.^48,49,50,51^ However, the social categories of race and ethnicity used in this study do reflect the underrepresentation of those socially defined groups and the genetic admixture that they represent in the knowledge base on hearing-loss genetics.^52^

Our results may have limited generalization for the general population, especially those who never sought care or were lost to follow-up; the patients in our study were those who sought and received care at a tertiary academic medical center. Although we report on a diverse population, our findings are still representative of the specific diversity found in the San Francisco Bay Area, align with the broad categorizations used in our medical records, and do not reflect the multiple subgroups that exist within each broad racial and ethnic category.

## Conclusions

The findings of this cohort study suggest that genetic testing is broadly useful in improving clinical management of children with HL. Those with unilateral HL, late-identified HL, more comorbidities, and from a URM group have lower genetic diagnosis rates; we did not find an association between diagnostic yield and age of identification, progression of HL, type of HL, or severity of HL. We also detailed the diagnostic yield in terms of the causative genes and their variants, showing in particular that nearly one-third of genetic diagnoses provided significant prognostic value in identifying progressive or syndromic associations. This knowledge may help improve patient counseling and shared decision-making for patients and families who are deciding whether to obtain genetic testing. More research is warranted to discover and characterize pathologic genes that result in HL for those who have been historically underrepresented in research and medicine as we continue to advance the care we provide for children with HL.

## Notes

Supplement. **eTable 1.** Description of Study Cohort (n = 426) by Year Tested **eTable 2.** Frequency of Individuals With Diagnostic Variants, Categorized by Ethnicity and Causative Genes
