# Supplementary material for: Outcomes of Gene Panel Testing for Sensorineural Hearing Loss in a Diverse Patient Cohort
Source: JAMA Netw Open. 2022 Sep 27;5(9):e2233441. doi: 10.1001/jamanetworkopen.2022.33441 (PMC9516276; doi:10.1001/jamanetworkopen.2022.33441)
Supplement: Supplement. — eTable 1. Description of Study Cohort (n = 426) by Year Tested eTable 2. Frequency of Individuals With Diagnostic Variants, Categorized by Ethnicity and Causative Genes [file jamanetwopen-e2233441-s001.pdf]

Supplementary Online Content

Liao EN, Taketa E, Mohamad NI, Chan DK. Outcomes of gene panel testing for sensorineural hearing loss in a diverse patient cohort. *JAMA Netw Open*. 2022;5(9):e2233441. doi:10.1001/jamanetworkopen.2022.33441

eTable 1. Description of Study Cohort (n = 426) by Year Tested

eTable 2. Frequency of Individuals With Diagnostic Variants, Categorized by Ethnicity and Causative Genes

This supplementary material has been provided by the authors to give readers additional information about their work.

© 2022 Liao EN et al. *JAMA Network Open.*

eTable 1. Description of Study Cohort (n = 426) by Year Tested

Demographics	2019, n=92<br><br>No. (%)	2020, n=201<br><br>No. (%)	2021, n=128<br><br>No. (%)
Age, median (range), years	7 (0-18)	7 (0-18)	10 (0-18)
Gender			
- Female	45 (48.9)	106 (52.7)	48 (37.5)
- Male	47 (51.1)	95 (47.3)	79 (61.7)
- Trans	0 (0)	0 (0)	1 (0.8)
Under-represented minority			
- URM	63 (68.5)	147 (73.1)	91 (71.1)
- Not URM	27 (29.4)	52 (25.9)	35 (27.3)
- Declined to state	2 (2.2)	2 (1.0)	2 (1.6)
Race/Ethnicity^			
- Hispanic	32 (34.8)	101 (50.3)	61 (47.7)
- Asian	13 (14.1)	30 (14.9)	22 (17.2)
- Black	5 (6.5)	18 (9.0)	10 (7.8)
- White	26 (28.3)	57 (28.4)	22 (17.2)
- Other	22 (23.9)	29 (14.4)	23 (18.0)
Primary language			
- English	56 (60.9)	116 (57.7)	79 (61.7)
- Spanish	22 (23.9)	57 (28.4)	34 (26.6)
- Mandarin, Cantonese	3 (3.3)	6 (3.0)	5 (3.9)
- American Sign Language	2 (2.2)	7 (3.5)	0 (0)
- Other language	9 (9.8)	15 (7.5)	10 (7.8)
Insurance			
- Private	31 (33.7)	45 (22.4)	35 (27.3)
- Public	61 (66.3)	155 (77.1)	92 (71.9)
- None	0 (0)	1 (0.5)	1 (0.8)
**Comorbidities**			
ICD-10 codes, median (range)	1 (0-25)	2 (0-40)	2 (0-25)
ICD-10 organ systems involved, median (range)	1 (0-5)	1 (0-9)	0 (1-10)
ASA score, mode (range)	2 (1-4)	2 (1-4)	2 (1-4)
**Audiologic Data**			
Age at identification			
- Early identification (failed NHS)	35 (38.0)	85 (42.3)	54 (42.2)
- Late identification (passed NHS)	34 (37.0)	56 (27.9)	38 (29.7)
- Late discovery (unknown NHS)	23 (25.0)	60 (29.9)	26 (28.1)
Characterization of HL			
- Progressive	28 (30.4)	41 (20.4)	28 (21.9)
- Stable	64 (69.6)	160 (79.6)	100 (78.1)
Laterality of HL			
- Unilateral	20 (21.7)	41 (20.4)	28 (21.9)
- Bilateral	72 (78.3)	160 (79.6)	100 (78.1)

© 2022 Liao EN et al. *JAMA Network Open.*

Type of HL*			
- Sensorineural	74 (80.4)	154 (76.6)	108 (84.4)
- Conductive	4 (4.4)	7 (3.5)	5 (3.9)
- Mixed	8 (8.7)	16 (8.0)	8 (6.3)
- Neural	6 (6.5)	8 (4.0)	4 (3.1)
- Unspecified	7 (7.6)	22 (11.0)	6 (4.7)
Severity of HL			
- PTA better ear, median (range), dB	30 (0-120)	30 (1-116)	34 (0-120)
- PTA worse ear, median (range), dB	43 (5-120)	46 (5-116)	45 (5-120)
**Genetic diagnosis**			
Received a genetic diagnosis	18 (19.6)	51 (25.4)	37 (28.9)
Did not receive a genetic diagnosis	74 (80.4)	150 (74.6)	91 (71.1)

5 patients were tested from 2015 to 2018. As this was such a small cohort, their demographic information was not included in this table to protect patient privacy.
*Does not add up to 100% because patients can identify as more than one category
ASA: American Society of Anesthesiologists Physical Classification System. HL: hearing loss. ICD-10: International Classification of Diseases 10th Revision. PTA: pure tone average. URM: under-represented minority.

© 2022 Liao EN et al. *JAMA Network Open.*

eTable 2. Frequency of Individuals With Diagnostic Variants, Categorized by Ethnicity and Causative Genes

	Hispanic	Asian	Black	White	Other
**Frequency**	205	96	37	134	91
**Diagnostic yield, n (%)**	40 (29)	33 (33)	6 (4)	31 (23)	25 (18)
**Diagnostic variants, n**	49	48	7	43	28
GJB2, 98					
- c.-23+1 G>A	1	1		1	
- c.34 G>T	1				
- c.35del	11	1	1	12	6
- c.35 G>T	1				
- c.101 T>C	3			1	
- c.109 G>A	3	32		1	6
- c.139 G>T	1	1		2	
- c.235delC		3			
- c.269 T>C	1			4	1
- c.283 G>A	1				
- c.313_326del14				1	
- Deletion involving Exon 2	1				
***STRC, CATSPER2,* 13**					
- 15q15.3 deletion	4		1	3	2
- Deletion of Exon(s) 19-24				1	
- Deletion Including Exons 19-29	1				1
***SLC26A4,* 9**					
- c.2 T>C			1		
- c.716 T>A	1				
- c.916dupG		1		1	
- c.1541 A>G			1		
- c.2027 T>A		1		1	
- c.2228 T>G		1		1	
***MYO7A,* 6**					
- c.321_322insA				1	
- c.494 C>T				2	
- c.721 C>T		1			
- c.5420_542dupAG		1			
- c.5660 C>T				1	

© 2022 Liao EN et al. *JAMA Network Open.*

***CDH23*, 5**					
- c2329_2330delAC				1	
- c.6050-15 G>A			1	1	
- c.6682delG					1
- c.9629_9632delTCAA				1	
***MYO15A*, 4**					
- c.3336delG				1	
- c.3385 C>T				1	
- c.4252 G>A	1				
- c.6509+5 G>A	1				
***USH2A*, 6**					
- c.13040_13062del23ins10		1			
- c.15089 C>A	1				
- c.2304 C>A				1	
- c.9570+1 G>A					1
- Deletion of Exon(s) 4	1			1	
***KCNQ4*, 3**					
- c.18dupG			1		
- c140 T>C					1
- c.835-1 G>T					1
***OTOGL*, 5**					
- c.919-1 G>A					1
- c.2472delA	2	2			
***GJB6*, 2**					
- 13q12.11 Deletion	2				
***ALMS1*, 2**					
- c.11768delA	1				
- c.3609 T>G	1				
***OTOF*, 2**					
- c.1966dupC	1				
- c.2225 T>C		1			
***OTOG*, 2**					
- c.2489_2490ins12	1				
- c.2701 G>T					1
***PDZD7*, 2**					
- c.561dupG	1				
- c.2089delG	1				
***SOX10*, 2**					
- c.-84-1 G>C					1

© 2022 Liao EN et al. *JAMA Network Open.*

- c.1137delC				1	
USH1C, 2					
- c.1458_1459del					2
***ADGRV1*, 1**					
- c.1547G>A					1
***EYA1*, 1**					
- c.880 C>T	1				
***MIR96*, 1**					
- r.12 G>A					1
***MITF*, 1**					
- c.710+1 G>A					1
OPA1, 1					
- c.556+2 T>G		1			
***OSBPL2*, 1**					
- c.180_181delCA	1				
***POU3F4*, 1**					
- c.765 G>A	1		1		
***PTPRQ*, 1**					
- c.1359+2 T>C	1				
***TMPRSS3*, 1**					
- c.208delC				1	
***WFS1*, 1**					
- c.124 C>T				1	
**Multigene deletion, 1**					
- 10p15.3p14 Deletion	1				

All children included. Blank cells indicate the frequency was zero.

© 2022 Liao EN et al. *JAMA Network Open.*
